# Corrigendum to “Role and mechanism of Circ‐PDE7B in the formation of keloid”

**DOI:** 10.1111/iwj.14869

**Published:** 2024-04-25

Tang Y, Li X. Role and mechanism of Circ‐PDE7B in the formation of keloid. *Int Wound J*. 2023;20(9):3738–3749. doi:10.1111/iwj.14269

Under ‘Materials and methods’, in the ‘Samples collection’ section, the text ‘XXX’ should be replaced by ‘Xi'an Central Hospital’.

In the ‘Author contributions’ section, the text should be replaced by ‘Yueling Tang designed and performed the research; Yueling Tang and Xiaojing Li analysed the data; Yueling Tang wrote the manuscript. All authors read and approved the final manuscript’.

We apologise for this error.

